# Single-cell and multi-omics analyses identify CAMP-associated neutrophil remodeling during radiochemotherapy in cervical cancer

**DOI:** 10.3389/fcell.2026.1773562

**Published:** 2026-03-09

**Authors:** Wenqian Li, Rui Ran, Jing Li

**Affiliations:** 1 The Department of Anesthesiology, Chongqing Health Center for Women and Children, Chongqing, China; 2 Women and Children’s Hospital of Chongqing Medical University, Chongqing, China

**Keywords:** CAMP, cervical cancer, neutrophils, radiochemotherapy, single-cell sequencing

## Abstract

**Background:**

To investigate key genes and immune microenvironment dynamics in cervical cancer progression and radiochemotherapy (RCT), focusing on cathelicidin antimicrobial peptide (CAMP)-mediated neutrophil regulation.

**Methods:**

Integrated transcriptomic data from TCGA, GTEx, and GEO were analyzed to identify differentially expressed genes associated with cervical cancer (CC) and RCT. Prognostic genes were selected via univariate and multivariate Cox regression. Single-cell RNA sequencing (scRNA-seq) characterized cellular composition, gene expression, and neutrophil subsets. CAMP-high and CAMP-low neutrophils were analyzed for differential expression, functional enrichment, pseudotime trajectories, and cell–cell communication.

**Results:**

Three prognostic genes (CAMP, CCDC116, and GLB1L3) were identified, with CAMP highly expressed in cervical cancer tissues but markedly downregulated after RCT. At the single-cell level, CAMP showed significant differential expression in tumor-associated and RCT-related neutrophils. Differential analysis and GO enrichment of neutrophils stratified by CAMP expression revealed enhanced innate immune activation, cytokine signaling, and granule secretion features following RCT. Pseudotime analysis demonstrated that CAMP expression gradually increased as neutrophils differentiated toward tumor-associated states, whereas it significantly decreased at terminal states after RCT. Cell–cell communication analysis further indicated that CAMP-high neutrophils exhibited strengthened signaling with fibroblasts and epithelial cells, particularly involving key ligands such as CXCL, COL1, and LAMC; RCT effectively suppressed tumor-specific inflammatory and extracellular matrix remodeling signals. *In vitro* experiments show that CAMP promotes malignant proliferation and activates inflammatory pathways in cervical cancer, whereas RCT suppresses these effects and modulates the COL1A1/COL1A2–CD44 and ANXA1–FPR1/FPR2 axes, reshaping tumor microenvironmental adhesion and immune activity.

**Conclusion:**

CAMP is a key regulator of neutrophil differentiation and tumor immune microenvironment remodeling in cervical cancer and during RCT. Its modulation of cell–cell communication networks suggest potential as a biomarker for treatment response and a therapeutic target.

## 1 Introduction

Cervical cancer (CC) is one of the most common malignancies in women worldwide, with over 600,000 new cases and approximately 340,000 deaths reported in 2020, imposing a particularly heavy burden in low- and middle-income countries (Ma et al., 2025; Son et al., 2025). Although widespread implementation of HPV vaccination and screening programs has partially reduced incidence, a high proportion of cases are still diagnosed at advanced stages, and the risks of recurrence and metastasis remain significant, posing substantial challenges to cervical cancer management (Gennigens et al., 2022; Rimel et al., 2022). Clinically, concurrent radiochemotherapy (RCT) has become the standard of care for locally advanced cervical cancer, significantly improving local control and survival outcomes (Cetina et al., 2006; Shrivastava et al., 2018). However, patients exhibit considerable variability in radiotherapy sensitivity, and a subset develops radioresistance, resulting in treatment failure. This suggests that cervical cancer RCT sensitivity is regulated by both intrinsic tumor cell signaling and multiple factors within the tumor microenvironment (TME) (Wu et al., 2023; Zhou et al., 2022).

Accumulating evidence indicates that cervical cancer initiation and progression are closely associated with the immune microenvironment, where the extent of immune cell infiltration, phenotypic plasticity, and dynamic changes in inflammatory mediators collectively determine tumor immune evasion and therapeutic response (Chen F. et al., 2022; Zou et al., 2021). TME remodeling not only contributes to tumor growth, invasion, and metastasis but also directly influences adaptation and resistance to therapeutic stress (Arneth, 2020). Notably, radiotherapy can induce persistent local inflammation, stromal fibrosis, hypoxia, and vascular damage, while simultaneously trigger immunosuppressive processes and altering the balance between pro- and anti-inflammatory signaling (Barker et al., 2015; Denk and Greten, 2022). Tumor cells can secrete pro-inflammatory factors such as IL-6, IL-1α, TGF-β, and TNF-α, driving the transformation of cancer-associated fibroblasts (CAFs) into inflammatory CAFs (iCAFs) (Hos et al., 2020), while radiotherapy further promotes secretion of various cytokines associated with radioresistance (Shevtsov et al., 2019). In addition, radiotherapy-induced hypoxia diminishes oxygen-dependent DNA damage effects and activates HIF-1 signaling, enhancing tumor cell survival (Semenza, 2004).

Immune cells are considered central regulators of TME remodeling following RCT. Studies have shown that regulatory T cells (Tregs) are significantly enriched in tumors after irradiation (Kachikwu et al., 2011). The CD4^+^CD25^+^Foxp3^+^ Treg population can suppress effector anti-tumor immunity through cell–cell contact and secretion of cytokines such as TGF-β, IL-4, and IL-10 (Chen et al., 2005; Shevach, 2002). Moreover, radiotherapy can upregulate PD-L1 on tumor cells, thereby impairing the cytotoxic activity of activated T cells and NK cells (Deng et al., 2014). Concurrently, radiotherapy may recruit a substantial number of tumor-associated neutrophils (RT-Ns), whose infiltration can drive sterile inflammation and activate tumor-specific cytotoxic T cells, contributing to tumor regression (Takeshima et al., 2016). Furthermore, RCT enhances the antitumor activity of effector T cells and activates tissue-resident memory T cells within the TME, thereby potentiating the efficacy of immune checkpoint inhibitors (Lauret et al., 2021). However, immune cells within the TME are not solely regulated by radiotherapy; their complexity and dynamic changes profoundly influence therapeutic outcomes (Wang et al., 2024). Radiotherapy can both induce the accumulation of immunosuppressive cells and activate other anti-tumor immune subsets, establishing a delicate balance (Grassberger et al., 2019; Guo et al., 2023; Wu et al., 2025). This balance is influenced not only by radiation dose and fractionation schedule (Gao et al., 2023), but also by tumor type, patient immune status, metabolism, and gene-specific therapeutic responses (Chen et al., 2025).

Therefore, systematically elucidating the key molecular and cellular regulatory networks that govern cervical cancer progression, immune microenvironment remodeling, and radiochemotherapy response holds substantial basic and clinical significance. This study aims to integrate multi-omics data from TCGA, GTEx, and GEO, combining differential gene screening, prognostic model construction, single-cell heterogeneity analysis, pseudotime trajectory modeling, and cell–cell communication network analysis to comprehensively identify key molecules associated with cervical cancer development and treatment response at both tissue and single-cell resolution. By systematically identifying and validating the responsive cell subpopulations, expression dynamics, and functional enrichment of key genes, this work provides potential core therapeutic targets for cervical cancer and radiochemotherapy.

## 2 Materials and methods

### 2.1 Data acquisition and processing

Cervical cancer (CC) transcriptome datasets used in this study were obtained from TCGA and GTEx, both derived from *Homo sapiens*. After integration, the datasets included tumor tissue samples from 306 CC patients and cervical tissue samples from 13 healthy individuals. Pre-RCT and post-RCT transcriptome datasets, GSE168009 and GSE56363, were downloaded from the GEO database (https://www.ncbi.nlm.nih.gov/geo/), also from *H. sapiens*. The integrated RCT datasets included tumor samples from 9 patients before RCT and 12 patients after RCT. When multiple probes were annotated to the same gene, the average expression value of those probes was calculated for downstream analysis.

### 2.2 Removal of batch effects

Prior to differential expression analysis, batch effects arising from samples of different origins were corrected using the remove Batch Effect function in the limma package in R, in conjunction with an appropriate design matrix. Principal component analysis (PCA) was then performed to visualize the data before and after correction, enabling assessment of the effectiveness of batch effect removal.

### 2.3 Differential expression analysis

Differential expression analysis and data processing were performed using R and relevant packages, including DESeq2, edgeR, data.table, and dplyr (Wang et al., 2022). Genes with |logFC| > 0.5 and p-value <0.05 were considered differentially expressed. Differential analyses were conducted for cervical cancer versus normal samples and RCT post-treatment versus pre-treatment samples. The intersection of the identified differentially expressed genes (DEGs) from these comparisons was used for subsequent analyses.

### 2.4 Survival analysis

Univariate Cox regression analysis of DEGs in cervical cancer versus normal samples was performed using the survival R package (Zhang X. et al., 2023). Genes with p-value <0.1 were selected, The caret package was used to randomly divide the sample dataset into a training set and a test set in a 7:3 ratio. Random forest analysis was performed on each set separately using randomForest. Genes were screened based on Coefficient >0, followed by multivariate Cox regression analysis to construct a prognostic model. Survival curves for the prognostic model were plotted using the survminer package. Samples were classified into high-risk and low-risk groups based on the median risk score.

The risk score for the prognostic model was calculated as follows: Risk score = −0.1792 × CAMP - 0.1199 × GLB1L3 - 0.3007 × CCDC116.

### 2.5 Single cell sequencing

The cervical cancer scRNA-seq dataset GSE208653 was downloaded from the GEO database. The data originates from *H. sapiens*, and the data type is high-throughput sequencing. Five samples were selected from this dataset: two normal cervical tissue samples and three cervical tumor tissue samples. The chemoradiotherapy-treated CC scRNA-seq dataset GSE236738 was also downloaded from the GEO database, with data sourced from *H. sapiens* and in high-throughput sequencing format. This dataset includes six samples: three pre-treatment cervical cancer tissue samples and three post-treatment cervical cancer tissue samples. Prior to single-cell analysis, quality control is essential to remove low-quality cell data and reduce noise, ensuring more reliable data analysis. The dataset employed the following quality control criteria to exclude low-quality cells: gene count per cell greater than 300 and less than 4,500, and mitochondrial gene expression ratio lower than 10%.

### 2.6 Dimensionality reduction, clustering, and cell annotation

The Seurat package (version 5.1.0) in R software (version 4.3.2) was used to integrate the CC scRNA-seq samples and the chemoradiotherapy-treated cervical cancer samples, creating Seurat objects for subsequent single-cell data processing (Liu et al., 2023). First, the NormalizeData function was used to standardize the raw data, followed by identification of highly variable genes, with a set number of 2000 genes. The decontX function was then applied to estimate and predict the level of contamination by ambient free RNA in each cell, and cells with contamination rates greater than 0.3 were excluded. Next, the RunHarmony function was employed to remove batch effects across all samples. The ScaleData function was used to scale the data and correct for the impact of mitochondrial-related gene expression. The top 2000 most variable genes across all samples were used as input for principal component analysis (PCA), performed using the RunPCA function (Zhang J. et al., 2023). The first 20 PCA dimensions were selected, followed by clustering analysis using the FindClusters function, which employs a Shared Nearest Neighbor (SNN)-based optimized clustering algorithm. A clustering tree was visualized and assessed using the clustree package (version 0.5.1) across same resolutions (Both are 0.4). Finally, clustering results were visualized using the RunUMAP function with the default parameters and the first 20 PCA dimensions. Cell annotation was performed by comparing the identified clusters to known lineage or cell type-specific marker genes, integrating findings from previous studies (Qu et al., 2023).

### 2.7 Expression of prognostic genes in single-cell data

Key prognostic genes Cathelicidin Antimicrobial Peptide (CAMP) were extracted from both single-cell datasets, and their expression levels were analyzed. Statistical significance was calculated using the rstatix package to identify in which cell types the expression of prognostic genes was significantly higher in the disease group compared to the normal group. Boxplots were generated using the ggpubr package for visualization.

### 2.8 Differential expression and enrichment analysis of key genes

The expression levels of CAMP genes in the disease group were extracted from key cell types in the single-cell data. Based on the average expression values of CAMP genes, cells were divided into high and low expression groups. Differentially expressed genes between the disease group and the normal group in each cell type were identified using the FindMarkers function with parameters logfc. threshold = 0.1 and min. pct = 0.1. Differential genes were selected based on the criteria of |logFC| > 0.5 and p-value <0.05. Gene Ontology (GO) and Kyoto Encyclopedia of Genes and Genomes (KEGG) enrichment analyses of the differential genes were performed using the clusterProfiler package.

### 2.9 Pseudotime analysis

Pseudotime analysis of key cell types was performed using the monocle2 package. A CellDataSet (cds) object was created using the newCellDataSet function, with the following input data: (1) the gene-cell matrix of raw count data from Seurat-processed scRNA-seq data, (2) meta. data from the single-cell data, which was converted into an S4 object using the new function, and (3) a data frame containing gene names, also converted into an S4 object using the new function. The expression data in the cds object was normalized, and genes expressed in fewer than 10 cells were filtered out. For trajectory analysis, high-variance genes identified using the VariableFeatures function were used for pseudotime ordering of cells. Dimensionality reduction was performed using the reduceDimension function with parameters reduction_method = “DDRTree” and max_components = 2. The cell trajectory was visualized and ordered using the plot_cell_trajectory function. Furthermore, we employed Spearman correlation analysis to evaluate the correlation between CAMP expression levels and pseudotime, and further utilized a generalized additive model (GAM) to model the nonlinear relationship between the two. By testing the significance of the smoothing term, we conducted a statistical assessment of the overall trend of CAMP expression changes with pseudotime.

### 2.10 Cell-cell communication analysis

To quantify the communication network between different cell types, we utilized the CellChat package to investigate potential cell communications. scRNA-seq data from the disease group were extracted, and cells were divided into high and low expression groups based on the average expression of CAMP genes. The standardized gene-cell matrix and cell annotation information for each group were extracted. A CellChat object was created using the createCellChat function, and the human ligand-receptor database provided by CellChat was imported. High-expression genes were identified, and corresponding ligand-receptor pairs were selected. These results were then used to construct a protein-protein interaction (PPI) network. The computeCommunProb function was employed to predict the communication probability between cells based on gene expression values. Subsequently, low-quality communication relationships between cells were filtered out using the filterCommunication function with the parameter min. cell = 10. Finally, CellChat objects for each group were merged, and the results were visualized using the netVisual_circle and netVisual_bubble functions.

### 2.11 Cell culture and model construction

HeLa and Ect1 cell lines were obtained from ATCC. All cell lines were confirmed to be free of *mycoplasma* contamination by the supplier. Cells were cultured in high-glucose DMEM supplemented with 10% fetal bovine serum (FBS) and 1% penicillin/streptomycin (PS) under standard conditions (5% CO_2_, humidified incubator) (Li et al., 2023). CAMP-overexpressing and CAMP-knockdown HeLa cell models were established using siRNA (purchased from GenePharma). The experimental groups were as follows: Blank control (Blank), CAMP overexpression (CAMP-OE), CAMP knockdown (CAMP-KD), radiochemotherapy group (RCT), radiochemotherapy plus CAMP overexpression (RCT + CAMP-OE), and radiochemotherapy plus CAMP knockdown (RCT + CAMP-KD).

Radiochemotherapy (RCT) was simulated by X-ray irradiation combined with cisplatin (DDP; Nuoxin, Jiangsu Hengrui). Briefly, cells were seeded in six-well plates and allowed to reach approximately 85%–90% confluence. Cells were then treated with DDP at a final concentration of 10 μM, followed by X-ray irradiation at doses of 0, 2, or 5 Gy for 30 min. After irradiation, cells were returned to the incubator and cultured for an additional 24 h before sample collection. Total RNA was extracted according to the manufacturer’s instructions using a rapid RNA extraction kit (Cat# RN001, ES Science, China).

### 2.12 Quantitative real-time PCR (qPCR)

Total RNA was isolated from Hela and Ect1 cells using TRIzol reagent following the manufacturer’s protocol. RNA quantity and quality were determined by spectrophotometry. Equal amounts of RNA were reverse-transcribed into cDNA using a commercial reverse transcription kit. qPCR was performed with SYBR Green Master Mix on a real-time PCR system. The cycling program included an initial denaturation followed by repeated denaturation and annealing/extension steps. Gene expression levels were normalized to an internal control and calculated using the 2^−ΔΔCt^ method.

### 2.13 Western blot assay

Ect1 and Hela cells (CAMP-OE, CAMP-KD) were collected and lysed in RIPA buffer containing protease and phosphatase inhibitors. Protein concentrations were determined using a BCA assay. Equal amounts of protein were separated by SDS–PAGE and transferred onto PVDF membranes (Zhai et al., 2023). After blocking with 5% non-fat milk or BSA, membranes were incubated with primary antibodies overnight at 4 °C, followed by HRP-conjugated secondary antibodies. Protein signals were visualized using an enhanced chemiluminescence system, and band intensities were quantified with ImageJ software. Target protein levels were normalized to appropriate loading controls.

### 2.14 Cell viability assay (CCK8)

Cells were seeded into 96-well plates at an appropriate density (3,000/hole) and allowed to adhere overnight. After the indicated treatments, 10 µL of CCK8 solution (Cat No. PF00004) was added to each well, followed by incubation at 37 °C for 1–4 h. Absorbance was measured at 450 nm using a microplate reader. Cell viability was calculated relative to untreated control cells.

### 2.15 Statistical analysis

All experiments were performed in at least three independent replicates. Continuous data are presented as the mean ± standard deviation (SD). Comparisons between two groups were conducted using an unpaired Student’s t-test. For comparisons among multiple groups, one-way analysis of variance (ANOVA) was applied, followed by Tukey’s *post hoc* test when homogeneity of variance was satisfied; otherwise, Welch’s ANOVA with Dunnett’s T3 *post hoc* test was used. Categorical variables were analyzed using the chi-square (χ^2^) test. Statistical analyses were carried out using GraphPad Prism, and a two-sided P value <0.05 was considered statistically significant.

## 3 Result

### 3.1 Integration of differential analysis and survival models to identify prognostic genes in cervical cancer with RCT treatment

**FIGURE 1:**
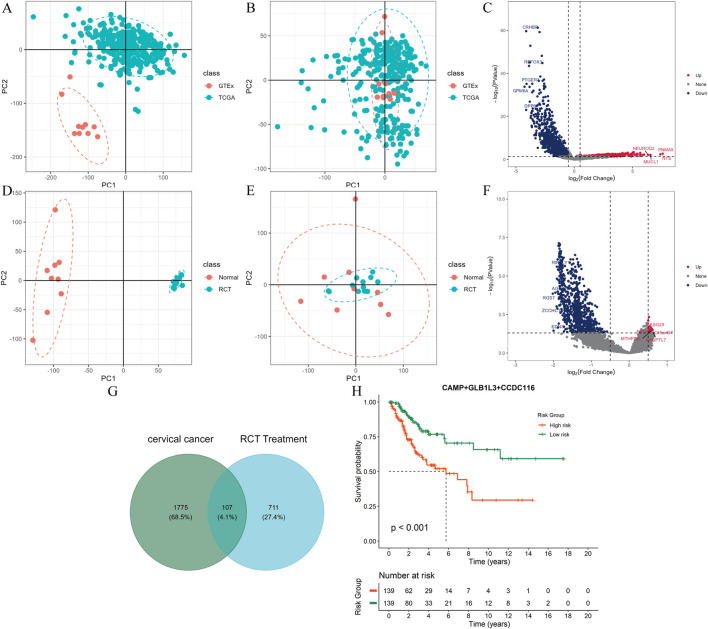
Transcriptomic data analysis and prognostic gene identification in CC and RCT treatment. **(A,B)** Correction of batch effects in the transcriptomic data of cervical cancer vs. normal group. **(C,D)** Correction of batch effects in the transcriptomic data of post-RCT vs. pre-RCT group. **(E,F)** Volcano plots of mRNA expression differences between cervical cancer vs. normal group and post-RCT vs. pre-RCT group. **(G)** Venn diagram showing the intersection of differentially expressed genes between cervical cancer vs. normal group and post-RCT vs. pre-RCT group. **(H)** Kaplan–Meier survival curves for CAMP, CCDC116, and GLB1L3.

To explore the key response molecules in CC and its chemoradiotherapy (RCT) treatment process, we integrated and performed differential analysis on the TCGA–GTEx (tumor vs. normal) and GSE168009/GSE56363 (pre-RCT vs. post-RCT) datasets. Principal component analysis (PCA) revealed significant batch effects in both merged datasets. After batch effect correction using the removeBatchEffect function, the batch effects were notably reduced (Figures 1A–D). Finally, 1882 differentially expressed genes (DEGs) were identified based on edgeR (|logFC| > 0.5, *P* < 0.05), of which 520 were upregulated and 1,362 were downregulated (Figure 1E). For the pre- and post-RCT data, limma analysis identified 818 DEGs, with 21 upregulated and 797 downregulated genes (Figure 1F).

To identify key genes associated with cervical cancer prognosis, we intersected the DEGs between CC vs. normal tissue and pre- and post-RCT treatment. A total of 107 common DEGs were identified (Figure 1G). Based on these 107 genes, univariate Cox regression analysis was performed on the cervical cancer dataset with a threshold of *P* < 0.1, resulting in 21 genes with potential prognostic value for further multivariate Cox analysis. Subsequently, we performed multivariate Cox regression analysis on the 21 genes and selected 7 independent prognostic-related genes based on a *P* < 0.05. To further assess their clinical significance, Kaplan–Meier survival curves were constructed based on the expression levels of each gene, dividing patients into high- and low-expression risk groups, and comparing overall survival (OS) between the two groups. The results indicated that only CAMP, CCDC116, and GLB1L3 showed significant survival differences between the high- and low-risk groups (*P* < 0.05).

We further developed a combined risk score model based on these 3 genes and reclassified patients into high-risk and low-risk groups according to the median value of the risk scores. Kaplan–Meier survival analysis revealed that patients in the high-risk group had significantly worse OS compared to the low-risk group (*P* < 0.001), and the C-index is 0.692, suggesting that these 3 genes have robust and strong prognostic predictive ability (Figure 1H). Therefore, we identified CAMP, CCDC116, and GLB1L3 as key prognostic biomarkers for further studies. Subsequently, we assessed the expression patterns of these three genes in cervical cancer versus normal tissues and in post-RCT versus pre-RCT samples. We found that CAMP was markedly upregulated in cervical cancer tissues—a finding consistent with the results validated in external datasets (Supplementary Figure S1)—and was significantly downregulated following RCT treatment. Therefore, based on this convergent evidence, CAMP was designated as the key gene in this study.

### 3.2 scRNA-seq analysis reveals cellular composition features associated with cervical cancer and RCT treatment

**FIGURE 2:**
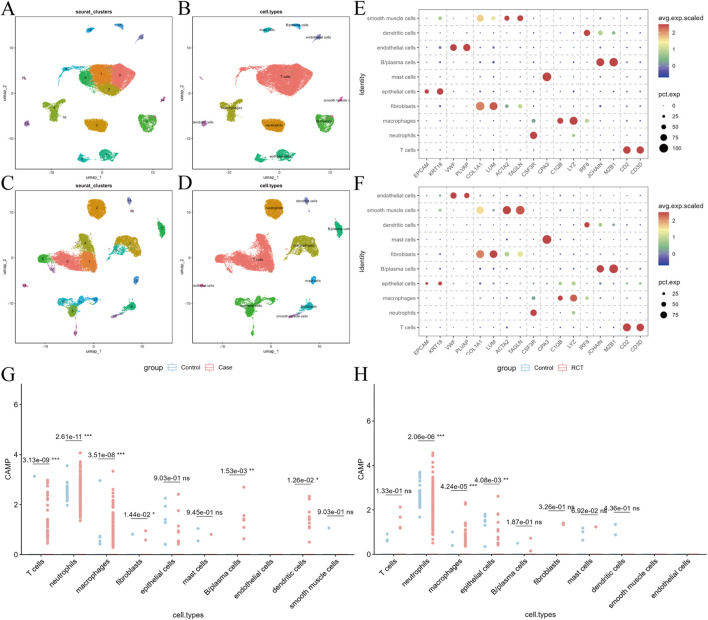
scRNA-seq data processing and analysis. **(A,B)** UMAP plots of cell clustering for the CC vs. normal group (resolution 0.5) and UMAP plots for the 10 cell types identified. **(C,D)** UMAP plots of cell clustering for the pre- and post-RCT treatment groups (resolution 0.4) and UMAP plots for the 10 cell types identified. **(E,F)** Expression bubble plots of marker genes for the 10 cell types in the CC vs. normal and pre-RCT vs. post-RCT groups. **(G,H)** Expression of CAMP in different cell subpopulations in the CC vs. normal and pre-vs. post-RCT groups.

Previous studies have shown that RCT can stimulate the remodeling of the tumor immune microenvironment (Lauret et al., 2021; Herter et al., 2023). To delineate CAMP expression dynamics across diverse immune and stromal cell populations and to identify the core cellular responders related to CC and RCT, we performed single-cell transcriptomic analyses on CC vs. normal tissues and on samples collected before vs. after RCT. After quality control, we retained 48,761 high-quality cells for the CC vs. normal comparison and 44,599 cells for the pre-vs. post-RCT comparison. In the CC vs. normal dataset, 17 clusters were identified at a resolution of 0.4 (Figure 2A) and annotated into 10 major cell types based on canonical marker genes (Figures 2B,E), including epithelial cells, endothelial cells, fibroblasts, smooth muscle cells, neutrophils, mast cells, macrophages, dendritic cells, B/plasma cells, and T cells. Using a resolution of 0.4, 20 clusters were identified in the pre-vs. post-RCT dataset and similarly classified into the same 10 major cell lineages (Figures 2C–F). Analysis of CAMP expression across cell populations showed that in the CC vs. normal comparison, CAMP was significantly altered in T cells, neutrophils, macrophages, fibroblasts, B/plasma cells, and dendritic cells, with the most pronounced difference observed in neutrophils (Figure 2G). In the RCT comparison, significant alterations in CAMP expression were detected in neutrophils, macrophages, and epithelial cells, again with neutrophils exhibiting the most prominent changes (Figure 2H). These findings indicate that CAMP displays notable expression variability across multiple immune and stromal cell lineages, and highlight neutrophils as the core cellular responders in both CC progression and RCT treatment.

Accordingly, we performed subcluster reclustering of neutrophils from both CC versus normal tissues and post-RCT versus pre-RCT samples, identifying three distinct subclusters (0–2). KEGG functional enrichment analyses were then conducted for each subcluster. The results demonstrated that, in both comparisons, all three subclusters were significantly enriched in immune regulatory pathways. Notably, subcluster 2 exhibited a stronger immune-activating signature, with prominent enrichment in pathways such as antigen receptor–mediated signaling, T cell receptor signaling, and immune response–activating cell surface receptor signaling pathways (Supplementary Figure S3). This further substantiates the central role of neutrophils in immune regulation.

### 3.3 Differential functional enrichment analysis of CAMP-associated neutrophils in CC and response to RCT

**FIGURE 3:**
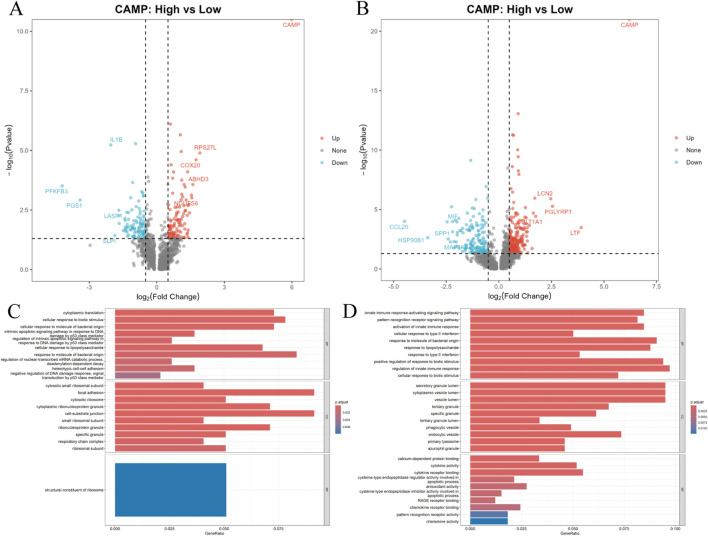
Differential analysis and enrichment of neutrophils. **(A,B)** Volcano plots of differential expression in neutrophils with high and low CAMP expression groups between cervical cancer (CC) and post-RCT treatment samples. **(C,D)** GO enrichment analysis of differentially expressed genes in neutrophils with high and low CAMP expression groups between cervical cancer and post-RCT treatment samples.

To further elucidate the key regulatory role of CAMP in CC and response to RCT, we extracted tumor-derived neutrophils from the CC vs. normal group (n = 2,923) and post-RCT treatment neutrophils (n = 3,160). After classifying the cells into high-expression and low-expression groups based on the mean expression of the key gene CAMP, we performed differential analysis and functional enrichment analysis. In the neutrophils derived from CC, a total of 201 CAMP-associated differentially expressed genes (|logFC| > 0.5, *P* < 0.05) were identified (Figure 3A). GO enrichment analysis of biological processes (BP) revealed that these genes were primarily involved in processes such as cytoplasmic translation, cellular response to biological stimuli, response to bacterial-derived molecules, regulation of the p53-mediated intrinsic apoptosis signaling pathway triggered by DNA damage, cellular response to lipopolysaccharides, and regulation of mRNA degradation by nuclear exosome complex (adenosine deaminase-dependent decay) (Figures 3B,C). Cellular component (CC) analysis highlighted enriched features such as cytoplasmic small ribosomal subunit, local adhesion, cytoplasmic ribosome, cytoplasmic ribonucleoprotein granules, cell-matrix junctions, small ribosomal subunit, ribonucleoprotein granules, and specific granules. The most significant molecular function (MF) category was ribosomal structural components, suggesting that high CAMP expression may drive enhanced protein synthesis and stress responses in tumor-associated neutrophils.

In the neutrophils post-RCT treatment, a total of 347 CAMP-associated differentially expressed genes were identified (Figure 3B). These genes were significantly enriched in biological processes related to the activation of innate immune responses, pattern recognition receptor signaling pathways, activation of type II interferon responses, responses to bacterial-derived molecules, responses to lipopolysaccharides, and positive regulation of biological stimulus responses (Figure 3D). Cellular component analysis revealed enrichment in immune effector structures such as secretory granule lumen, cytoplasmic vesicle lumen, vesicle lumen, tertiary granules, specific granules, tertiary granule lumen, phagocytic vesicles, and endocytic vesicles. MF analysis highlighted significant enrichment in key molecular functions, including calcium-dependent protein binding, cytokine activity, cytokine receptor binding, cysteine-type endopeptidase regulatory activity involved in apoptosis, antioxidant activity, cysteine-type endopeptidase inhibitory activity involved in apoptosis, RAGE receptor binding, and chemokine receptor binding. In summary, these results indicate that the level of CAMP expression not only defines the tumor-associated functional state of neutrophils in cervical cancer but also marks the immunological remodeling process induced by RCT. This suggests that CAMP plays a central driving role in both cervical cancer progression and treatment response.

### 3.4 Pseudotime analysis

**FIGURE 4:**
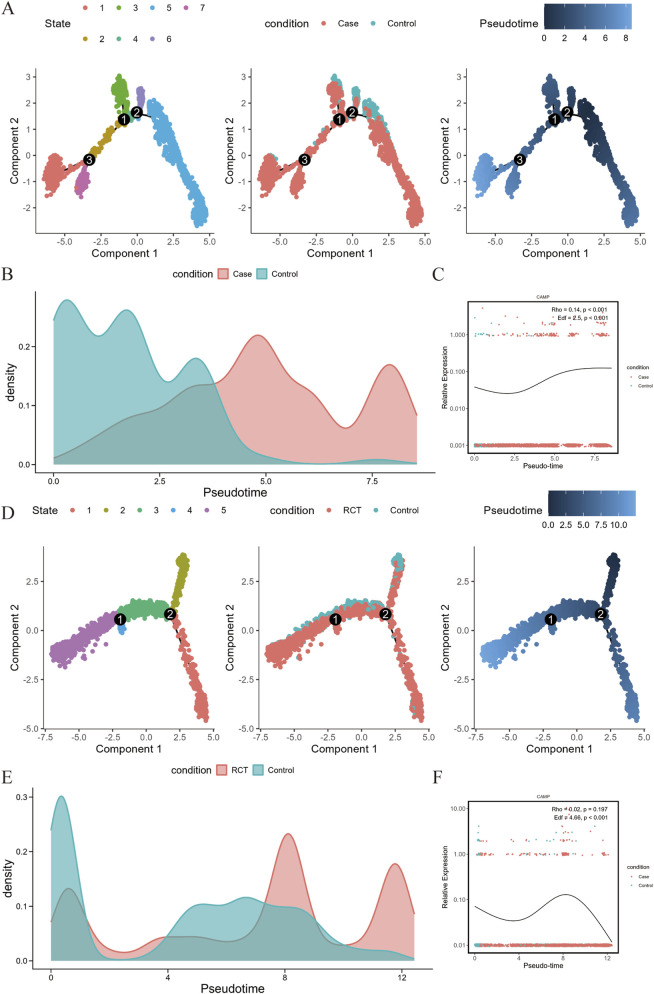
Pseudotime analysis of single-cell neutrophils. **(A)** The left plot shows the 7 differentiation states of neutrophils from the CC vs. normal group. The middle plot displays the distribution of normal and disease cells along the differentiation trajectory, while the right plot indicates the starting point of differentiation (darkest color) and the endpoint (lightest color). **(B)** Changes in cell density along pseudotime for normal and disease neutrophils from the CC vs. normal group. **(C)** Expression dynamics of the CAMP gene along pseudotime in neutrophils from the CC vs. normal group. **(D)** The left plot shows the 7 differentiation states of neutrophils from the pre- and post-RCT treatment groups. The middle plot displays the distribution of normal and disease cells along the differentiation trajectory, while the right plot indicates the starting point of differentiation (darkest color) and the endpoint (lightest color). **(E)** Changes in cell density along pseudotime for normal and disease neutrophils from the pre- and post-RCT treatment groups. **(F)** Expression dynamics of the CAMP gene along pseudotime in neutrophils from the pre- and post-RCT treatment groups.

To explore the potential regulatory role of CAMP in the differentiation states of neutrophils during cervical carcinoma progression and response to RCT, we performed pseudotime analysis on neutrophils from the CC vs. normal group and pre- and post-RCT treatment groups. In the CC vs. normal group, we extracted 4,117 neutrophils and performed dimensionality reduction and clustering (FindNeighbors and FindClusters functions using the first 20 PCA dimensions, which is shown in Supplementary Figure S3, FindClusters resolution 0.5). Pseudotime analysis revealed that neutrophils were classified into 7 differentiation states along the developmental trajectory, starting from normal cells and gradually transitioning to tumor-associated terminal states (Figure 4A). Notably, CAMP expression levels showed a significant positive correlation with pseudotime at the single-cell level, the expression of CAMP decreased in the early pseudotime stages but significantly increased in the later differentiation stages (Spearman Rho = 0.14, *P* < 0.001, Figures 4B,C), suggesting that CAMP plays a key role in the late-stage activation and functional remodeling of tumor-associated neutrophils. We further employed the Generalized Additive Model (GAM) to evaluate its nonlinear trend, with results demonstrating that CAMP expression exhibited significant dynamic changes along a pseudo-time series (EDF = 2.5, *P* < 0.001) and peaked during the late differentiation phase, suggesting that CAMP may be closely associated with the neutrophil differentiation process.

In the pre- and post-RCT treatment data, we extracted 4,274 neutrophils and performed dimensionality reduction and clustering with the same parameters. Pseudotime analysis divided the cells into 5 differentiation states, with the trajectory showing a transition from the pre-treatment state to the post-treatment state (Figure 4D). CAMP expression showed a weak positive correlation with pseudotime, but the correlation was not statistically significant (Spearman Rho = 0.02, *P* = 0.197, Figures 4E,F), indicating no clear overall linear trend. Further analysis using the Generalized Additive Model (GAM) revealed a significant dynamic change in CAMP expression along the pseudotime axis (*P* < 0.001). The expression gradually declined and approached zero during the late differentiation phase, suggesting that CAMP may exhibit stage-specific regulatory characteristics in the differentiation of neutrophils associated with RCT. In conclusion, CAMP demonstrates a dynamic expression pattern closely associated with neutrophil differentiation during both cervical cancer progression and RCT treatment, further supporting its potential biological significance as a tumor progression driver and a therapeutic response biomarker.

### 3.5 CAMP-high neutrophils reshape cell–cell communication networks in cervical cancer during chemoradiotherapy

**FIGURE 5:**
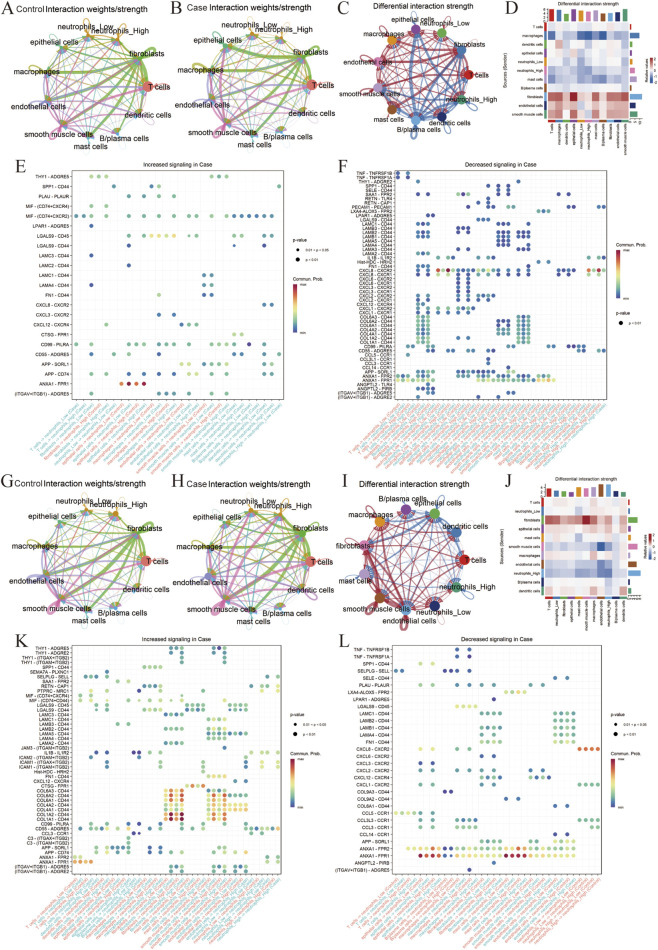
Single-cell cell communication analysis. **(A)** Cell communication strength between various cell types in the normal group (Control). **(B)** Cell communication strength between various cell types in the cervical cancer group (Case). **(C)** Differential communication weight network between cervical cancer (Case) and normal (Control) groups, with red indicating upregulation and blue indicating downregulation. **(D)** Heatmap of cell communication differences between cervical cancer and normal groups. **(E,F)** Upregulated and downregulated ligand-receptor pairs in the cervical cancer group. **(G)** Cell communication strength between various cell types in the pre-RCT treatment group (Control). **(H)** Cell communication strength between various cell types in the post-RCT treatment group (Case). **(I)** Differential communication weight network between post- and pre-RCT treatment groups, with red indicating upregulation and blue indicating downregulation. **(J)** Heatmap of cell communication differences between post- and pre-RCT treatment groups. (**K,L**) Upregulated and downregulated ligand-receptor pairs in the post-RCT treatment group.

To systematically characterize the cell–cell communication features of CAMP-high neutrophils during cervical cancer progression and treatment response, we first extracted single-cell transcriptomic data from cervical cancer tissues and normal cervical tissues, which were defined as the disease and control groups, respectively. Based on the average expression level of CAMP in neutrophils, these cells were further stratified into CAMP-high (neutrophil_High) and CAMP-low (neutrophil_Low) subsets, followed by cell–cell communication analysis. In both the disease and control groups, neutrophil_High exhibited the strongest communication weights with fibroblasts, indicating a potentially critical role of CAMP-high neutrophils in interactions with stromal cells (Figures 5A,B). We next compared the global communication patterns between the disease and control groups and found that the most prominent differences occurred between neutrophil_High and epithelial cells (Figure 4C). Specifically, compared with the control group, outgoing signals from neutrophil_High to epithelial cells were markedly reduced in the disease group, whereas incoming signals from epithelial cells to neutrophil_High were significantly enhanced (Figures 5C,D), suggesting a cancer-associated rewiring of communication directionality. At the ligand level, CTSG, LAMC2,ANGPTL2,CCL14,PLAU showed significant differences between the two groups. Further integrated analysis of communication signals revealed that CTSG, LAMC2, LAMC3, MIF, and PLAU were upregulated in the disease group, while ligands such as ANGPTL2, CCL14, CCL3, CCL3L1, and CCL5 were downregulated in the disease group (Figures 5E,F).

We further investigated single-cell datasets obtained before and after chemoradiotherapy (RCT), defining pre-treatment samples as the control group and post-treatment samples as the experimental group. Using the same stratification strategy based on CAMP expression, neutrophils were divided into CAMP-high and CAMP-low subsets, followed by communication analysis. Consistent with the above findings, neutrophil_High maintained the strongest communication weights with fibroblasts both before and after RCT, indicating a stable interaction pattern under therapeutic pressure (Figures 5G,H). Comparative analysis between post-RCT and pre-RCT samples revealed that the most pronounced communication changes again involved neutrophil_High and epithelial cells, with overall interactions between these two cell types being reduced after treatment (Figures 5I,J). At the ligand level, TNF, SELE, PLAU, THY1, SEMA7A, and SAA1 showed marked differences between the two conditions. Specifically, C3, CD55, CD99, COL1A1 and COL1A2were significantly increased in post-RCT samples (Figures 5K,L), whereas ANGPTL2,CCL14,CCL3L3,CCL5 and PLAU were markedly decreased after treatment (Figures 5M,N). Notably, before and after RCT treatment, the ligand–receptor interactions from other cell types to neutrophils with high or low CAMP expression exhibited significant differences. In particular, the COL1A1/COL1A2–CD44 ligand–receptor pair was markedly enhanced, whereas the ANXA1–FPR1/FPR2 interactions were significantly attenuated.

### 3.6 *In vitro* experiments preliminarily validate CAMP as a key response molecule in cervical cancer radiotherapy and chemotherapy

**FIGURE 6:**
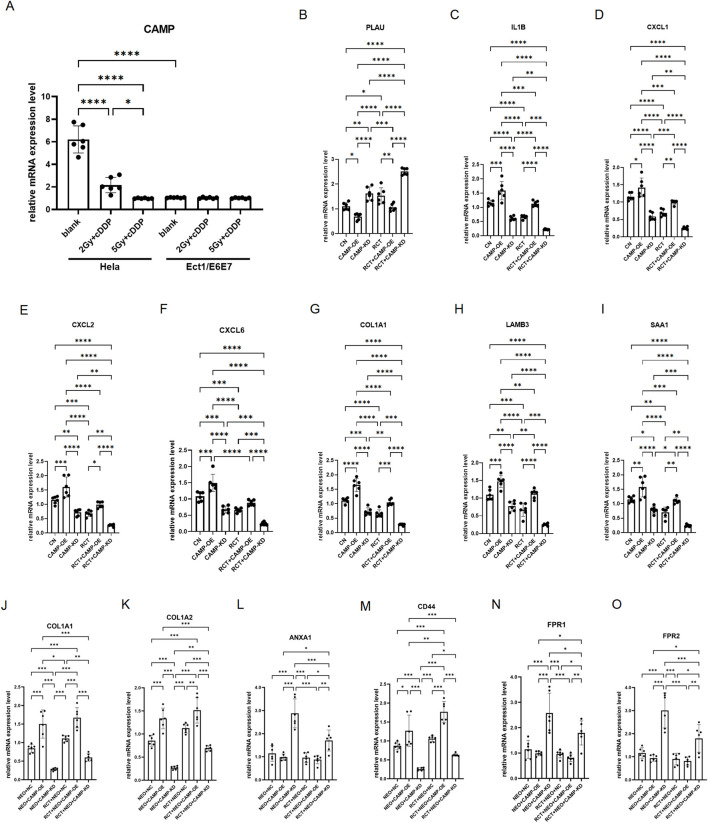
*In vitro* validation of key genes and associated ligand signaling involved in the response to chemoradiotherapy in cervical cancer. **(A)** mRNA expression of CAMP in normal cervical squamous epithelial cells from the external cervical os (Ect1/E6E7) and the cervical cancer cell line HeLa following chemoradiotherapy (RCT) treatment. RCT was simulated by X-ray irradiation at graded doses (Gy) for 30 min, followed by cisplatin (DDP) treatment for 24 h. **(B–I)** mRNA expression levels of differentially regulated ligand signals in HeLa cells from the Blank, CAMP overexpression (CAMP-OE), CAMP knockdown (CAMP-KD), RCT, RCT + CAMP-OE, and RCT + CAMP-KD groups. **(J,K)** mRNA expression levels of key ligand–receptor pairs in neutrophils and HeLa cells after co-culture before and after RCT treatment. Expression of the ligands COL1A1 **(J)**, COL1A2 **(K)**, and ANXA1 **(L)** was measured in HeLa cells, whereas expression of the receptors CD44 **(M)**, FPR1 **(N)**, and FPR2 **(O)** was assessed in neutrophils.

To determine whether CAMP functions as a key driver of cervical cancer and a critical molecular target mediating the response to radiochemotherapy, we performed *in vitro* functional validation using normal cervical squamous epithelial cells from the external cervical OS (Ect1/E6E7) and the cervical cancer cell line HeLa. Radiochemotherapy (RCT) was simulated by X-ray irradiation at graded doses combined with cisplatin (DDP) treatment. Quantitative PCR analysis demonstrated that CAMP expression was significantly elevated in HeLa cells compared with Ect1/E6E7 cells. Notably, RCT treatment markedly suppressed CAMP transcription in HeLa cells in a dose-dependent manner with increasing X-ray intensity (Figure 6A, *P* < 0.05). These findings suggest that upregulation of CAMP may promote cervical cancer progression and that CAMP represents a key molecular target of radiochemotherapy in cervical cancer.

Given that prior cell–cell communication analyses revealed significant differences in multiple key ligands—including PLAU, SAA1, CXCL6, CXCL2, CXCL1, IL1B, and COL1A1—between cervical cancer and normal tissues, as well as before and after RCT treatment, we subsequently validated the expression of these ligands at the cervical cancer cell level to determine whether their signaling is regulated by CAMP expression and RCT. The results showed that, compared with the control (CN) group, PLAU expression was upregulated in HeLa cells with CAMP overexpression as well as in CAMP knockdown cells, and PLAU levels were further significantly increased following RCT treatment regardless of CAMP expression status (Figure 6B, *P* < 0.05). In contrast, the other seven ligands—SAA1, CXCL6, CXCL2, CXCL1, IL1B, LAMB3, and COL14A1—exhibited an opposite expression pattern, being upregulated upon CAMP overexpression and downregulated following CAMP knockdown, while their expression was consistently suppressed after RCT treatment (Figures 6C–I,P < 0.05). The transcriptional changes of these key ligands were generally consistent with their expression patterns in epithelial cells—the primary cell type of origin in cervical cancer—before and after RCT treatment, with the exception of SAA1 and PLAU (Supplementary Figure S4). Collectively, these findings indicate that CAMP may promote cervical cancer progression and contribute to chemoradiotherapy resistance by regulating the expression of multiple key ligands and thereby reshaping the intercellular communication network within the tumor microenvironment.

Furthermore, cell–cell communication analysis indicated that key ligand–receptor axes within the immune network, including COL1A1/COL1A2–CD44 and ANXA1–FPR1/FPR2, were significantly altered in cervical cancer before and after RCT, supporting dynamic immune–tumor cell interactions in the tumor microenvironment. To validate these findings, we co-cultured CAMP-overexpressing or -knockdown Hela cells with neutrophils and subjected the system to RCT. PCR analysis was performed to assess the expression of the key receptors CD44 and FPR1/FPR2 in neutrophils and their corresponding ligands COL1A1/COL1A2 and ANXA1 in Hela cells, to determine whether RCT drives intercellular signaling dynamics. The results showed that RCT significantly enhanced COL1A1/COL1A2–CD44 signaling (Figures 6J,K,M, *P* < 0.05) while markedly suppressing ANXA1–FPR1/FPR2 signaling (Figures 6L,N,O, *P* < 0.05), with these effects being more pronounced under CAMP knockdown. These findings are consistent with the prior cell–cell communication analysis, suggesting that COL1A1/COL1A2–CD44 and ANXA1–FPR1/FPR2 axes represent critical ligand–receptor pairs mediating neutrophil immunoregulation in response to RCT in cervical cancer.

### 3.7 Functional effects of CAMP overexpression and knockdown on cervical cancer cell proliferation and inflammatory responses before and after RCT treatment

**FIGURE 7:**
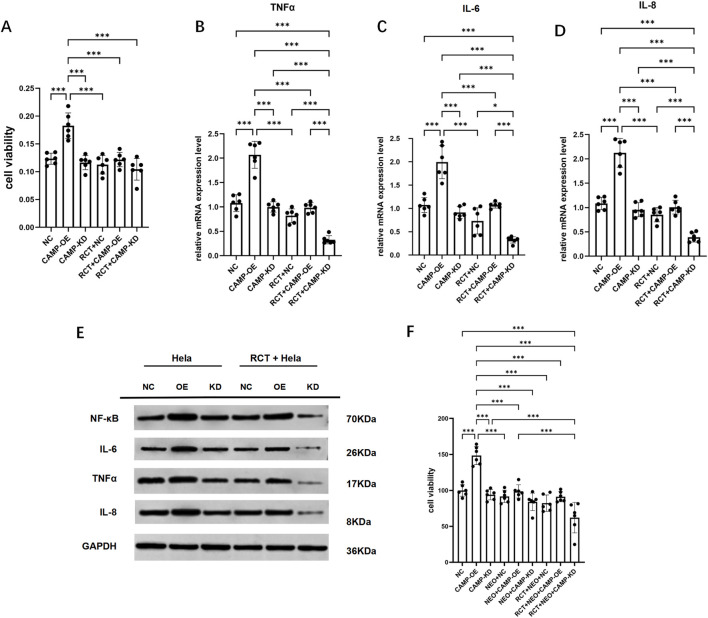
Functional effects of CAMP on cervical cancer cell proliferation and inflammatory responses under RCT treatment. **(A)** CCK-8 assay measuring cell viability in NC, CAMP-OE, and CAMP-KD HeLa cells before and after RCT treatment. **(B–D)** RT–qPCR analysis of mRNA expression levels of inflammatory cytokines TNFα **(B)**, IL-6 **(C)**, and IL-8 **(D)** in NC, CAMP-OE, and CAMP-KD HeLa cells before and after RCT treatment. **(E)** Western blot analysis of protein expression levels of NF-κB, TNFα, IL-6, and IL-8 in NC, CAMP-OE, and CAMP-KD HeLa cells before and after RCT treatment. **(F)** CCK-8 assay measuring the viability of HeLa cervical cancer cells in a neutrophil–HeLa co-culture system before and after RCT treatment.

To determine whether CAMP influences the functional phenotype of cervical cancer cells and whether RCT treatment can counteract CAMP-mediated effects, we first assessed the proliferative capacity of HeLa cells with CAMP overexpression or knockdown using the CCK-8 assay. CAMP overexpression significantly promoted HeLa cell proliferation, whereas CAMP knockdown suppressed it. Notably, RCT treatment inhibited cell proliferation in both the CAMP-overexpression and CAMP-knockdown groups (Figure 7A, *P* < 0.05). In addition, functional enrichment analyses suggested that RCT induces immune remodeling in cervical cancer (Figures 3C,D). Consistent with this, we examined the expression of inflammatory cytokines in HeLa cells with different CAMP expression levels before and after RCT treatment. RT–qPCR and Western blot analyses showed that CAMP overexpression significantly increased the mRNA and protein levels of TNFα, IL-6, and IL-8 (Figures 7B–D, *P* < 0.05), and activated the NF-κB inflammatory pathway (Figure 7E). Importantly, RCT treatment reversed the CAMP-induced inflammatory phenotype, suggesting that CAMP promotes a pro-inflammatory state in cervical cancer cells, whereas RCT attenuates this effect. In the neutrophil–HeLa co-culture system, we observed that neutrophils suppressed the enhanced proliferative capacity induced by CAMP overexpression, with an even more pronounced inhibitory effect when CAMP was knocked down (Figure 7C, *P* < 0.05). Following RCT treatment, the suppression of HeLa cell proliferation in the co-culture system became even stronger. These findings suggest that neutrophils may inhibit the malignant proliferative phenotype of cervical cancer cells through the secretion of cytokine (Wang et al., 2025), and that RCT further enhances this neutrophil-mediated anti-tumor activity.

## 4 Discussion

Cervical carcinoma (CC) continues to represent a major global health burden, with persistently increasing incidence and mortality rates (Bray et al., 2018). Although radiochemotherapy (RCT) remains the standard treatment for locally advanced CC, its clinical efficacy varies substantially among patients, implying that intrinsic tumor characteristics and the tumor microenvironment (TME) critically shape treatment outcomes (Sun, 2016; Wang et al., 2011; Xu et al., 2025). Consequently, this study aimed to identify key molecular drivers and potential regulatory mechanisms involved in CC initiation and progression, TME remodeling, and responses to radiotherapy. Through integrative analyses of differential gene expression and survival modeling, we identified CAMP, CCDC116, and GLB1L3 as candidate genes associated with CC prognosis (Figure 1). Among them, CAMP emerged as particularly noteworthy, as it was significantly upregulated in CC tissues and markedly downregulated following RCT. This expression pattern suggests that CAMP may function as a key oncogenic driver and a potential mediator of treatment response.

CAMP (LL-37), the active peptide derived from hCAP18, has been implicated in diverse tumor-promoting processes across multiple malignancies. Previous studies have demonstrated that LL-37 enhances MAPK signaling through ErbB2 to promote breast cancer cell proliferation and migration (Weber et al., 2009); similarly, myeloid-derived LL-37 activates Wnt/β-catenin signaling to stimulate lung tumor growth (Ji et al., 2019). In ovarian cancer, CAMP facilitates tumor progression by recruiting bone marrow–derived mesenchymal stem cells, thereby enhancing angiogenesis and immunosuppression within the TME (Coffelt et al., 2009; Piktel et al., 2016). Interestingly, LL-37 has also been shown to augment the antitumor effects of CpG-ODN by activating NK cells and boosting interferon signaling (Chuang et al., 2009), highlighting its context-dependent and multifaceted immunomodulatory roles (Chuang et al., 2009). These studies collectively suggest that CAMP plays a complex regulatory role in cancer biology, further supporting its potential as a key driver of cervical cancer progression and a critical target for RCT therapy.

Subsequently, we utilized single-cell sequencing to identify the key cellular subsets expressing CAMP before and after RCT in cervical cancer. Our analysis revealed that neutrophils were the primary cell type expressing CAMP in CC tissues and following RCT (Figures 3C,D). As an antimicrobial protein, CAMP is an essential component of the innate immune system (Li et al., 2006; Murakami et al., 2004; Turner et al., 1998; Wang, 2008; Woloszynek et al., 2012). It binds to bacterial lipopolysaccharides (LPS) (Li et al., 2006; Wang, 2008) and promotes the release of CXCL2 via neutrophil N-formyl peptide receptors, playing a critical role in immune defense (Woloszynek et al., 2012). Neutrophils’ ability to phagocytize and kill bacteria and fungi is a vital aspect of innate immunity (Turner et al., 1998), indicating a significant association between CAMP and neutrophil function in the context of cancer. Moreover, LL-37 has been shown to stimulate the release of exosomes from mouse bone marrow neutrophils (Kumagai et al., 2020), enhancing their antimicrobial potential. These findings suggest that CAMP-expressing neutrophils represent a core cell population in both the pathogenesis of cervical cancer and the immune response to RCT therapy. Pathway enrichment analyses (Figures 2G,H) revealed that CAMP overexpression markedly reshapes the functional states of neutrophils within the tumor microenvironment (TME). In tumor tissues, high CAMP expression is closely associated with sustained activation of pathways related to protein synthesis, cellular stress responses, and inflammation. Following radiochemotherapy (RCT), neutrophils exhibit enhanced innate immune activation, increased cytokine signaling, and elevated granule secretion capacity. These findings suggest that CAMP may serve as a key regulator mediating the functional reprogramming of neutrophils during tumor progression and therapeutic response.

Building on these observations, we further characterized the molecular actions of CAMP through *in vitro* experiments. CAMP not only upregulated the expression of inflammatory cytokines such as TNFα, IL-6, and IL-8, but also activated NF-κB–mediated inflammatory signaling pathways (Figures 7B–E). Notably, RCT partially reversed the CAMP-induced inflammatory activation and pro-proliferative phenotype (Figures 7A–E). Collectively, our findings indicate that CAMP promotes tumor progression by driving neutrophil inflammatory activation, whereas RCT exerts immunomodulatory and suppressive effects in this process. Further analysis using pseudotime trajectory and cell communication analysis revealed that CAMP-expressing neutrophils play a unique role in cervical cancer progression and RCT response, acting as central regulators in tumor microenvironment cell-to-cell communication remodeling. In CC tissues, CAMP-overexpressing neutrophils not only enhance interactions with fibroblasts and epithelial cells to construct a tumor-specific inflammatory network but also regulate matrix remodeling via key molecules such as CXCL2. RCT treatment significantly reversed this aberrant communication pattern, restoring some normal tissue signaling. This finding is consistent with previous studies that suggest tumor-associated neutrophils can promote malignancy through ECM remodeling, chemokine amplification, and immune suppression (Chen Y. et al., 2022; Kim and Bae, 2016). In contrast, in the initial state, these neutrophils are genetically or therapeutically polarized into an anti-tumor phenotype, thereby exerting anti-tumor effects (Kwak and Houghton, 2025). Notably, the dynamic changes in CAMP expression and its strong correlation with neutrophil differentiation trajectories further support its dual biological function as both a tumor progression driver and a therapeutic response biomarker.

Based on the multi-omics analysis, we performed preliminary *in vitro* experiments to determine whether CAMP functions as a central regulator of the response to radiochemotherapy (RCT) in cervical cancer and its associated ligand signaling. Quantitative PCR analysis revealed that CAMP expression was significantly higher in normal cervical squamous epithelial cells (Ect1/E6E7) than in cervical cancer HeLa cells, whereas RCT treatment markedly suppressed CAMP transcription in HeLa cells (Figure 6A). These findings indicate that aberrant CAMP expression is closely associated with cervical cancer progression and suggest that CAMP may represent a potential molecular target influencing sensitivity to radiochemotherapy. Given that cell–cell communication analysis revealed significant differences in multiple key ligands within epithelial cells between cervical cancer and normal tissues, as well as before and after RCT (Supplementary Figure S4). To validate these findings, we examined the expression of these ligands in Hela cells. The results showed that CXCL6, CXCL2, CXCL1, IL1B, LAMB3, and COL14A1 were markedly upregulated in CAMP-overexpressing Hela cells and significantly downregulated upon CAMP knockdown (Figures 6B–I). Notably, the expression of these CAMP-associated ligands was suppressed following RCT, suggesting their potential involvement in the antitumor effects of the treatment.

In the neutrophil–Hela co-culture system, two major ligand–receptor axes exhibited treatment-dependent regulation: COL1A1/COL1A2–CD44 signaling was significantly enhanced following RCT, whereas ANXA1–FPR1/FPR2 signaling was markedly inhibited (Figures 5K,L; Figures 6J–O). The former is associated with extracellular matrix remodeling and cell adhesion (Li et al., 2026; Xu et al., 2024; Zhu et al., 2025), whereas the latter typically mediates pro-inflammatory chemotaxis and immune activation (Jeong and Bae, 2020; Kelly et al., 2022; Schloer et al., 2019). This bidirectional regulatory pattern indicates that RCT may not only act directly on tumor cells but also reshape the communication network between epithelial cells and neutrophils, thereby modulating the post-treatment immune microenvironment. Collectively, these findings suggest that CAMP may influence the immune response to RCT by regulating epithelial ligand profiles and neutrophil communication axes. These findings not only provide new insights into the mechanisms underlying cervical cancer progression and RCT response but also suggest potential therapeutic targets for improving RCT efficacy. By targeting CAMP-expressing neutrophils or their associated signaling pathways, it may be possible to disrupt the tumor-promoting inflammatory network and restore normal tissue signaling, thereby enhancing the anti-tumor effects of RCT. Moreover, the dynamic changes in CAMP expression during tumor progression and RCT response highlight its potential as a biomarker for monitoring treatment efficacy and predicting patient prognosis. Future studies should further explore the precise mechanisms by which CAMP regulates neutrophil differentiation and function, as well as its interactions with other cell types in the tumor microenvironment.

## 5 Conclusion

This study identifies CAMP as a key regulator of neutrophil remodeling and the tumor microenvironment in cervical cancer and its response to radiochemotherapy. High CAMP expression drives tumor progression and inflammatory signaling, while radiochemotherapy reverses these pro-tumor effects and reshapes intercellular communication. CAMP emerges as a potential prognostic biomarker and a promising therapeutic target for enhancing radiochemotherapy efficacy.

## Data Availability

The original contributions presented in the study are included in the article/Supplementary Material, further inquiries can be directed to the corresponding author.
