# Supplementary material for: Single-cell and multi-omics analyses identify CAMP-associated neutrophil remodeling during radiochemotherapy in cervical cancer
Source: Front Cell Dev Biol. 2026 Mar 9;14:1773562. doi: 10.3389/fcell.2026.1773562 (PMC13006679; doi:10.3389/fcell.2026.1773562)
Supplement: Supplementary file 1 [file DataSheet1.docx]

**Supplementary Figures**

**
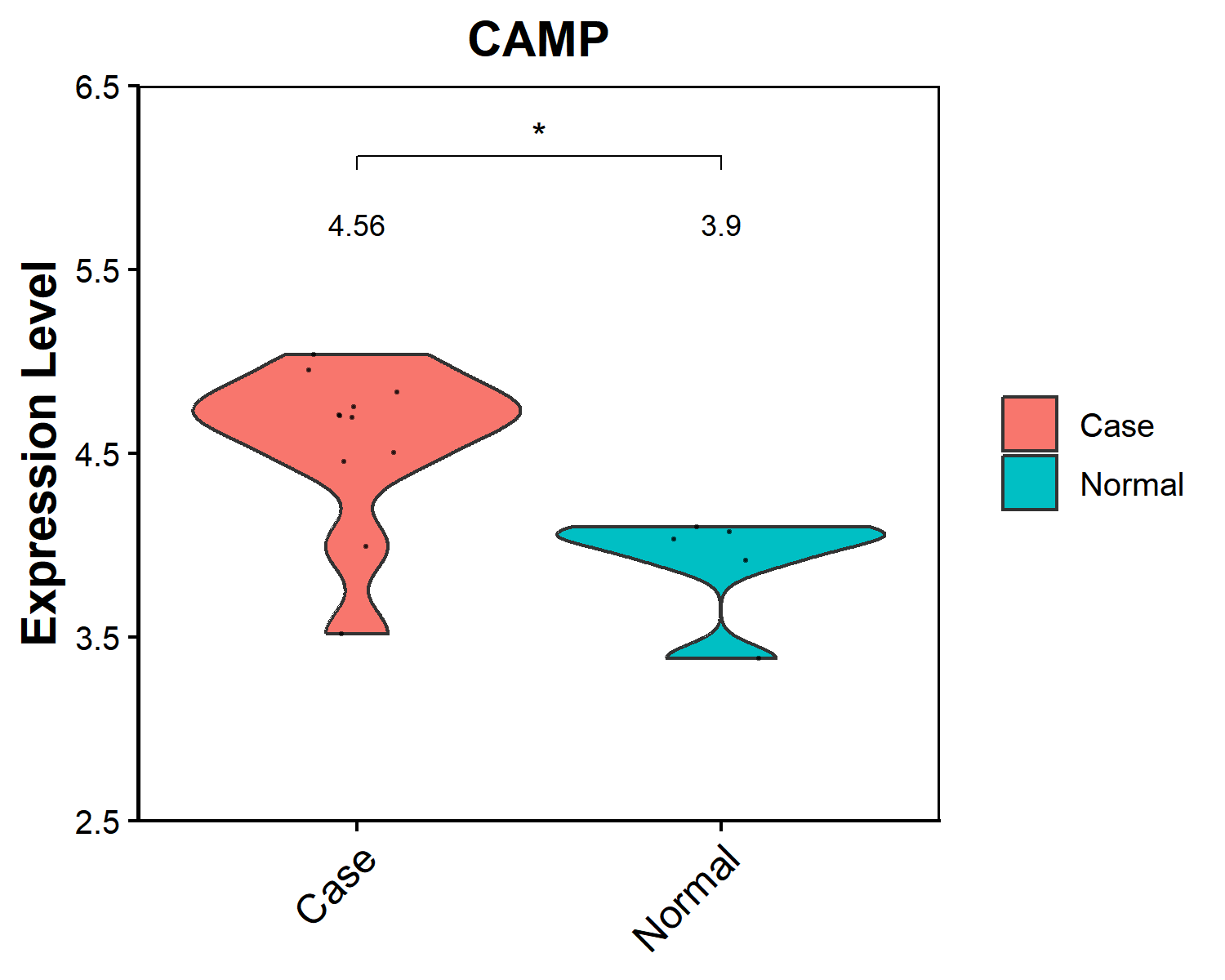
**

**Figure S1** | Validation of CAMP expression differences between normal tissue and cervical cancer using external transcriptomic datasets.

**
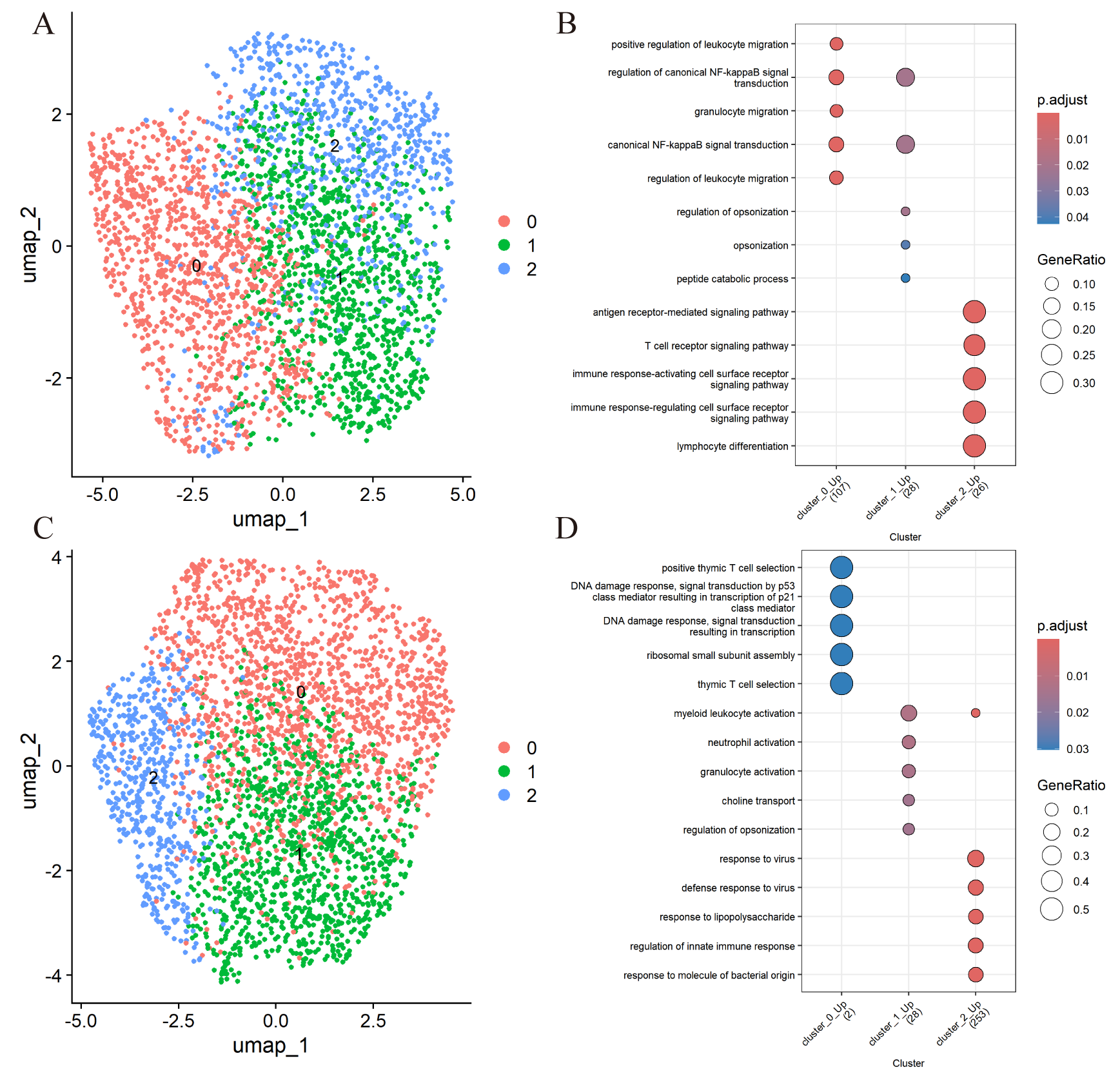
**

**Figure S2** | Reclustering of neutrophil subpopulations and KEGG functional enrichment of the subclusters. A-B: UMAP plots showing the reclustering of neutrophils in normal tissue vs. cervical cancer (A) and pre-RCT vs. post-RCT (C), divided into three clusters (0–2). C-D: Bubble plots of KEGG functional enrichment analysis for cervical cancer vs. normal tissue (B) and post-RCT vs. pre-RCT (D).

**
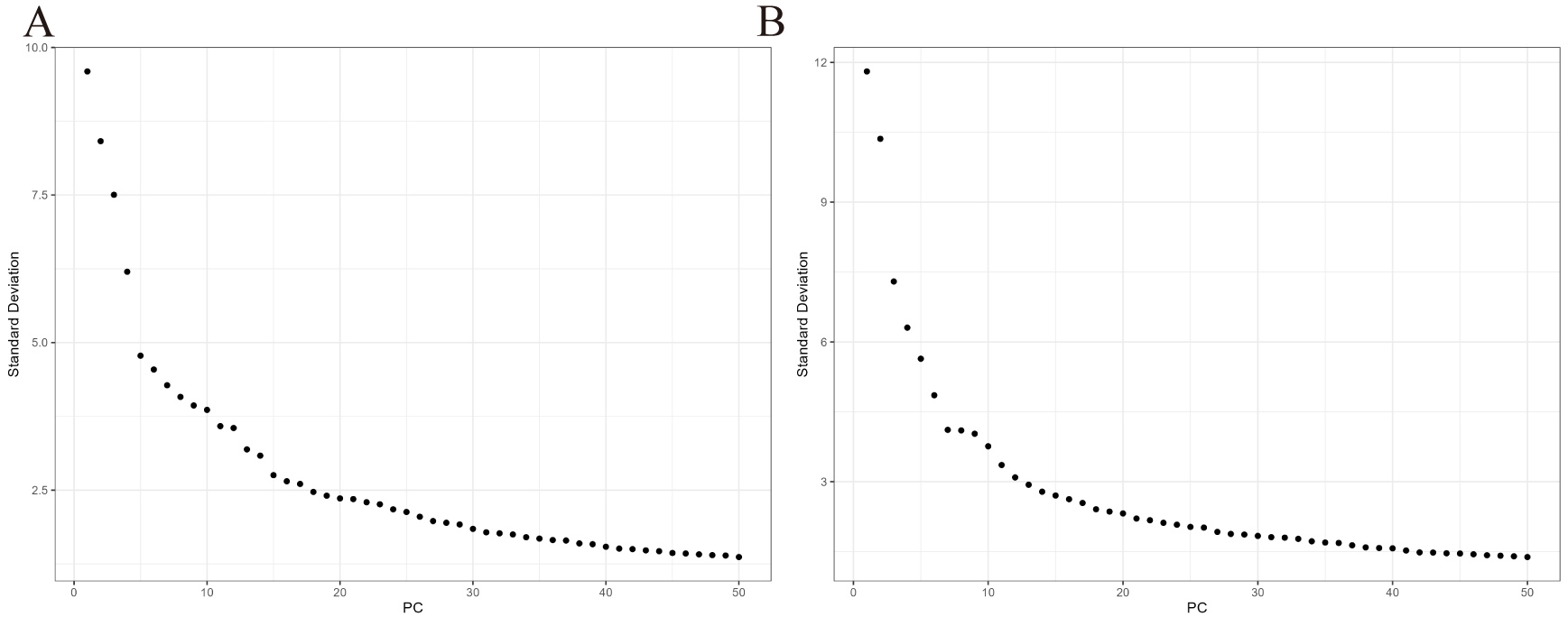
**

**Figure S3** | the Elbowplot of CC (A) and RCT (B). In the downstream dimensionality reduction and clustering analyses, we selected the top 20 principal components based on the elbow plot and the cumulative proportion of explained variance. The elbow plot indicated that the incremental gain in explained variance plateaued after approximately 20 components; therefore, these principal components were chosen for subsequent analyses.


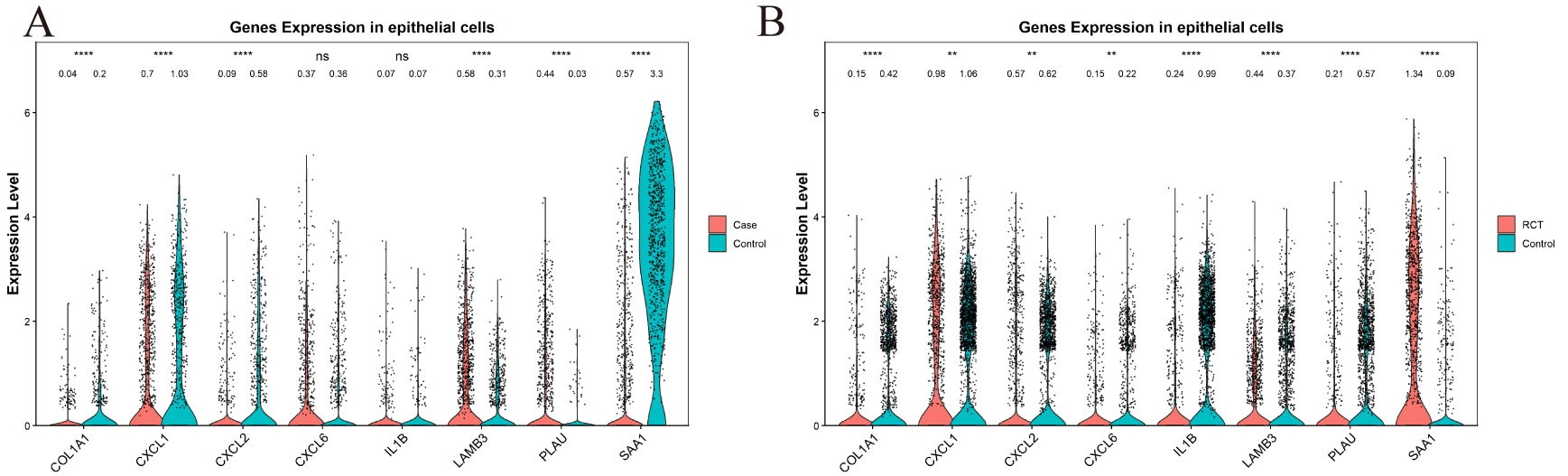


**Figure S4** | Differential expression of key ligands in epithelial cells. Expression differences of key ligands PLAU, SAA1, CXCL6, CXCL2, CXCL1, IL1B, and COL1A1 in epithelial cells between normal tissue and cervical cancer (A); expression differences of the same key ligands in epithelial cells before and after RCT treatment (B).
